# Vitamin D supplementation improved physical growth and neurologic development of Preterm Infants receiving Nesting Care in the neonatal Intensive Care Unit

**DOI:** 10.1186/s12887-023-04075-1

**Published:** 2023-05-20

**Authors:** Wei-qin Tang, Ning Ma, Li-ying Meng, Ya-wen Luo, Ying-jie Wang, Di Zhang

**Affiliations:** 1 Neonatology Department, The First Hospital of Qinhuangdao, No. 258, Wenhua Road, Haigang District, Qinhuangdao, 066000 Hebei China; 2 Endocrine Department, The First Hospital of Qinhuangdao, Qinhuangdao, Hebei China

**Keywords:** Nesting, Vitamin D supplementation, Preterm infants, Physical growth, Neurologic development

## Abstract

**Objective:**

To study the effects of vitamin D supplementation on physical growth and neurologic development of very preterm infants receiving nesting intervention in the neonatal intensive care unit (NICU).

**Methods:**

A total of 196 preterm infants had been hospitalized in NICU with the gestational age (GA) between 28 and 32 weeks. Among them, 98 preterm infants received nesting intervention, and the other 98 cases received both nesting and vitamin D supplementation (400 IU). The interventions were continued until 36 weeks postmenstrual age (PMA). The 25(OH)D serum levels, anthropometric parameters, and Premie-Neuro (PN) scores were compared at 36 weeks PMA.

**Results:**

Higher median serum level of 25(OH)D was found in the nesting + vitamin D [38.40 ng/mL (IQR: 17.20 ~ 70.88) ng/mL] as compared to the nesting group [15.95 ng/mL (IQR: 10.80 ~ 24.30) ng/mL] at 36 weeks PMA. Besides, infants receiving combined nesting intervention and vitamin D supplementation had less proportion of vitamin D deficiency [VDD, 25(OH)D levels < 20 ng/mL] than those receiving nesting intervention alone. After intervention, the anthropometric parameters of infants, including weight, length, BMI and head circumference were improved in the nesting + vitamin D group as compared to the nesting group at 36 weeks PMA, with higher scores of neurological, movement and responsiveness.

**Conclusions:**

Vitamin D supplementation effectively decreased the prevalence of VDD and led to higher concentrations of 25(OH)D at 36 weeks PMA. This was one more study that supported the necessity of vitamin D supplementation to improve physical growth and neurologic development of preterm-born newborns who received nesting intervention in the NICU.

## Introduction

Globally, 1 child in 10 is born prematurely every year, namely before completing 37 weeks of gestation, and the premature birth as a risk factor for healthy growth and development has been a trigger for acute and chronic health conditions [1, 2]. Except for a history of clinical fragility and social vulnerability, children born within the range of prematurity tend to have developmental and growth problems, such as school difficulties, worse motor repertoire, bad behavior problems and growth alterations [2, 3]. Neonates are abruptly separated from their mother after birth in the neonatal intensive care unit (NICU), an unnatural habitat, to warrant for survival, which often culminated in negative disparities for long-term child health (e.g., mental, physical and emotional health) [4]. During their stay in the a high-tech NICU environment, the infants receive external stimuli, such as light, intense noise, non-aggregated painful interventions [5]. Therefore, more interventions have been reported to promote the comfort of preterm infants and to minimize damage caused by exposure to stress in NICU, such as Kangaroo care [4], auditory intervention (white noise, recorded mother’s voice, and MiniMuffs) [6], and Yakson and gentle human touch [7].

Positioning has been reported to be one of the most studied interventions among preterm infants through providing comfort and reducing stress, and nesting is a useful tool to promote a proper positioning of the preterm infant by some studies described recently [8, 9]. In brief, the nest resembles the maternal uterus by a cloth rolled in an “U” or “O” shape [5], which favored a more flexed posture for total containment of the baby’s movements from head to foot and facilitated alignment of head in relation to the body, thus improving neurobehavioral and muscle development of preterm infants [10].

Vitamin D storage received by newborns depends on 50–70% of the maternal 25-hydroxyvitamin D [25(OH)D] levels [11]. Because of maternal vitamin D supply deprivation during gestation, many preterm infants have low vitamin D stores [12]. The vitamin D deficiency (VDD) is closely correlated with increased risk of many diseases in the premature infants’ population, such as impaired immune function, pulmonary function deficits, impaired neurodevelopment and reduction of bone mass [13]. Thus, some experts have suggested a new approach to a higher intake of vitamin D supplementation (400 ~ 1000 IU/day) in preterm infants [11, 14, 15].

So, we aimed to investigate the effectiveness of vitamin D supplementation on physical growth and neurologic development for preterm infants receiving nesting care by nurse at NICU. We retrospectively enrolled 196 preterm newborns between 28 ~ 32 weeks’ gestational age (GA). Among them, 98 preterm infants received nesting intervention (nesting group), and the other 98 cases received combined nesting and vitamin D supplementation (nesting + vitamin D group). The 25(OH)D serum levels, anthropometric parameters, and Premie-Neuro (PN) score were compared at 36 weeks postmenstrual age (PMA).

## Methods and materials

### Subjects

Very preterm infants (GA < 32 weeks) required hospitalization in NICU as demonstrated by previous studies [16, 17]. A consecutive sample of preterm infants admitted to the NICU between May 2019 and May 2021 with the gestational age (GA) between 28 and 32 weeks were reviewed (n = 353). GA was determined by either self-reported information on the 1st day of the last menstrual period or ultrasound examination in the 1st trimester screening [18, 19]. The study was performed in accordance with the Declaration of Helsinki and approved by the Ethics Committee of the First Hospital of Qinhuangdao. The need for informed consent of parents and/or legal guardians was waived as a retrospective analysis.

### Inclusion and exclusion criteria

Inclusion criteria were: (1) having no central nervous system (CNS) disorders, including brain hemorrhage, syndromes with neurological impairment, history of seizure, and hypertonia; (2) having no brain abnormalities or major congenital/chromosomal anomalies; (3) having no gastro-intestinal, lung or kidney disease, such as necrotizing enterocolitis (NEC), gastrointestinal malabsorption, a major congenital anomaly, a congenital pulmonary or airway disorder, acute kidney injury and so on. We will exclude the following infants: (1) those born at > 32 weeks of gestation; (2) those with vomiting or regurgitation in less than 24 h, or a history of apnea within the last 72 h [5, 9], (3) those with bone fractures or injuries; (4) those with disorders of calcium metabolism; (5) those with no expectation of survival in first 2 weeks.

### Intervention

**Fig. 1:**
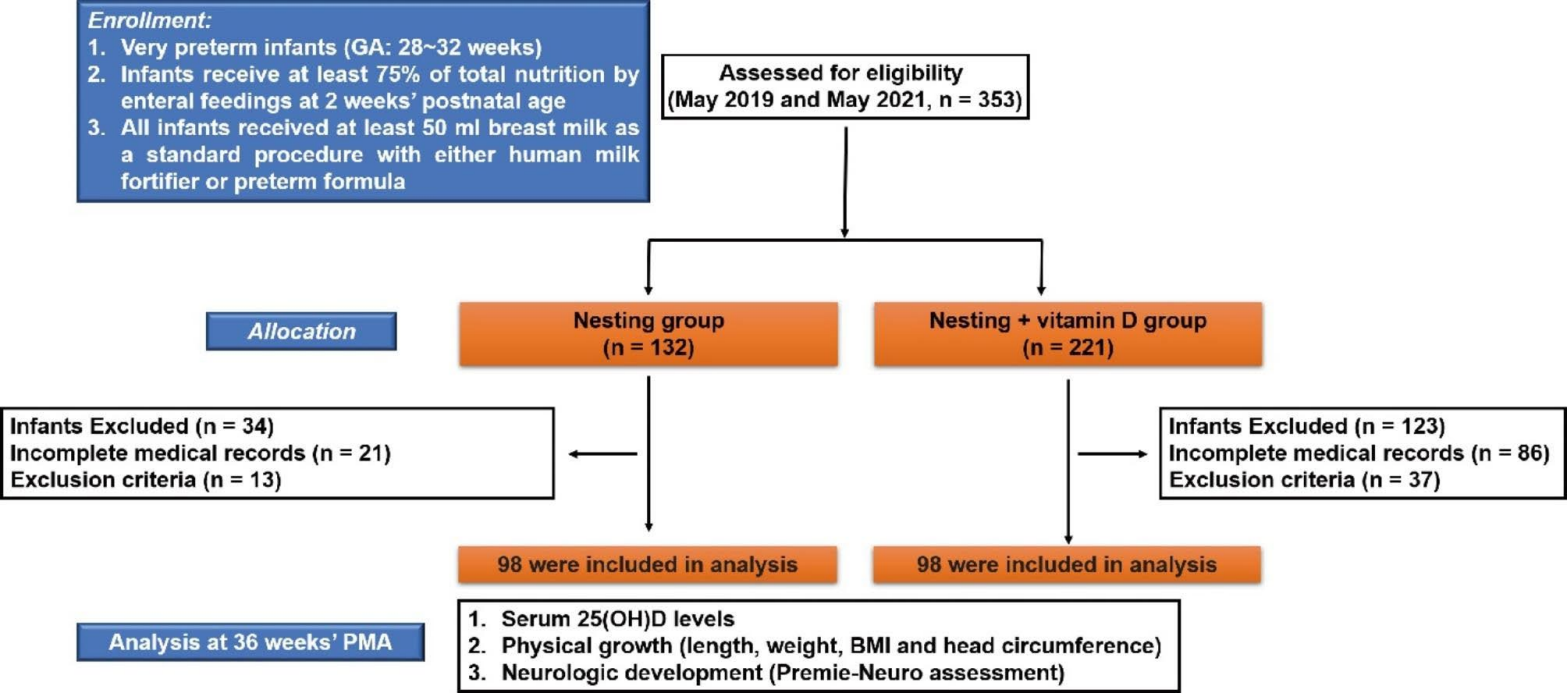
A CONSORT flow diagram in this retrospective study

A CONSORT flow diagram in this retrospective study was illustrated in Fig. 1. A total of 196 who could receive at least 75% of total nutrition by enteral feedings at 2 weeks’ postnatal age were finally enrolled in this analysis. The infants in the nesting group (n = 98) and nesting + vitamin D group (n = 98) were all laid in an O-shaped nest made of cotton cloth with a strip of cloth attached to the nest in supine position, which had perfect size for infants’ body. In order to make sure of recommended minimum vitamin D intake (200 to 300 IU/day) [12, 20], all infants received at least 50 ml breast milk as a standard procedure with either human milk fortifier (Vitamin D intake for infants weighing 1.5 kg: 283 ~ 320 IU/d) or preterm formula (Vitamin D intake for infants weighing 1.5 kg: 288 ~ 300 IU/d) until an enteral feeding volume of 160 ml/kg/day was reached. Except for nesting intervention, the preterm newborns in the nesting + vitamin D group were given 400 IU/d of vitamin D_3_ supplementation (Shandong DYNE Marine Biopharmaceutical Co., Ltd, China) once daily through orogastric tube or oral. The intervention was started in the first postnatal week of infants after blood sampling and continued until 36 weeks postmenstrual age (PMA). Pathologies related to prematurity were noted [21], such as late onset sepsis (LOS), hyaline membrane disease (HMD), bronchopulmonary dysplasia (BPD, classified as mild, moderate and severe [22]), intraventricular hemorrhage (IVH), periventricular leukomalacia (PVL) and retinopathy (ROP).

### Detection of 25(OH)D in serum

Serum 25(OH)D was assayed by an autoanalyzer (Savant-100, Beijing Savant Biotechnology Co., Ltd, China) using a chemiluminescent tracer. Vitamin D deficiency (VDD) was defined as serum 25(OH)D levels < 20 ng/mL [23].

### Physical growth at 36 weeks’ PMA

Measurements of length, head circumference, and weight were performed by bedside nurses. Naked weight was measured on a calibrated digital body weight scale. Length was measured in a supine position with a recumbent measuring board. Body mass index (BMI) was calculated from measured weight and length. The head circumference was measured with a self-retracting.

### Standardized neurologic assessment of the preterm infant

The neurological examination for preterm infants from 23 to 37 weeks PMA was assessed by a neonatologist (W.Q.T.) using the Premie-Neuro (PN), a reliable and valid neurologic assessment tool (Table 1). It consisted of 3 factors for a total of 24 items, including neurological subscale (8 items: arm recoil, arm traction, palmar grasp, plantar grasp, scarf sign, popliteal angle, heel to ear, movement type), movement subscale (8 items: tremors, thrashing, facial grimace, startle, yawn, color change, arm movements, leg movements), and responsiveness subscale (8 items: arm flexion, head lag, held sit, posterior neck, anterior neck, alert, ventral suspension, responsiveness) [24, 25]. Each of the items was assigned a score of 1, 3, or 5, with a total possible score ranging from 24 to 120. Internal consistencies (the Cronbach α) of these categories were 0.75 (neurological), 0.73 (movement), and 0.82 (responsiveness), respectively.

**Table 1:** Standardized neurologic assessment of the preterm infant by the Premie-Neuro (PN)

PN categories (a score of 1, 3, or 5 for each item, and total score ranging from 24 to 120)		
Neurologic (8 items)	Movement (8 items)	Responsiveness (8 items)
Arm recoil	Tremors	Arm flexion
Arm traction	Thrashing	Head lag
Palmar grasp	Facial grimace	Held sit
Plantar grasp	Startle	Posterior neck
Scarf sign	Yawn	Anterior neck
Popliteal angle	Color change	Alert
Heel to ear	Arm movements	Ventral suspension
Movement type	Leg movements	Responsiveness

### Data analysis

Statistical analysis was performed using GraphPad Prism Software (version 6, San Deigo, CA, USA), with statistical significance set at a two-sided *P* < 0.05. After Shapiro-Wilk analysis, the continuous data with normal distribution [presented as means ± standard deviations (SD)] were analyzed using independent t student, and those without normal distribution [presented as median and interquartile range (IQR, 25th ~ 75th percentile)] using Mann-Whitney test. Frequencies for categorical variables reported as n (%) between the 2 groups were determined by Fisher’ s exact test. Post hoc power analysis by G*Power 3.1.9.2 program was performed using appropriated statistical test (Proportions: Inequality, two independent groups, Fisher’s exact test) by the difference in the proportion of infants with VDD in nesting group (71.43%) and nesting + vitamin D group (34.69%) at 36 weeks’ PMA. Statistical power was 99.99% as calculated with α error = 0.5, showing that the sample size was adequate in this study.

## Results

### Baseline characteristics of preterm neonates between the two groups

As illustrated in Table 2, the demographic data of premature infants in this retrospective study was compared between the nesting and nesting + vitamin D groups. There were 98 infants with a mean GA of 29.65 (1.141) weeks in nesting group and 98 infants with a mean GA of 29.53 (1.262) weeks in nesting + vitamin D group, showing no statistical difference (*P* = 0.477). Furthermore, Independent t‑test showed no significant difference in the mean of maternal age, maternal serum 25(OH)D, parenteral feeding (days) and Apgar score at 5 min between the two groups (*P* > 0.05). The mode of delivery and the infant sex of the study population were also comparable, as well as the enteral feeding type and Vitamin D_3_ delivery (all *P* > 0.05). These results mentioned above indicated that these two groups were similar in terms of variables. Moreover, the incidence of comorbidities and days of hospital stay did not differ in the nesting and nesting + vitamin D groups (all *P* > 0.05).

**Table 2:** The demographic data of preterm infants participating in the study

Variables	Nesting group (n = 98)	Nesting + vitamin D group (n = 98)	*P*
Maternal age (years)	28.0 (24.75 ~ 35.0)	31.0 (23.75 ~ 37.0)	0.453
Gestational age (GA, weeks)	30.0 (29.0 ~ 31.0)	29.5 (28.0 ~ 31.0)	0.429
Maternal serum 25(OH)D (ng/ml)	10.60 (6.18 ~ 14.08)	9.80 (5.93 ~ 13.63)	0.645
Parenteral feeding (days)	9 (7 ~ 12)	10 (7 ~ 12)	0.591
Enteral feeding type [n (%)]			
Breast milk + human milk fortifier	71 (72.45%)	73 (74.49%)	
Breast milk + preterm formula	27 (27.55%)	25 (25.51%)	0.872
Vitamin D_3_ delivery [n (%)]			
Orogastric tube	36 (36.73%)	29 (29.59%)	
Oral	62 (63.27%)	69 (70.41%)	0.363
Apgar score at 5 min	7 (6 ~ 8)	7 (6 ~ 8)	0.341
Gender [n (%)]			
Male	48 (48.98%)	51 (52.04%)	
Female	50 (51.02%)	47 (47.96%)	0.775
Type of delivery [n (%)]			
Cesarean birth	85 (86.73%)	87 (88.78%)	
Vaginal	13 (13.27%)	11 (11.22%)	0.828
Clinical complications [n (%)]			
Bronchopulmonary dysplasia (BDP)	24 (24.49%)	20 (20.41%)	0.572
Hyaline membrane disease (HMD)	65 (66.33%)	57 (58.16%)	0.302
Retinopathy (ROP)	8 (8.16%)	4 (4.08%)	0.372
Periventricular leukomalacia (PVL)	7 (7.14%)	5 (5.10%)	0.767
Intraventricular hemorrhage (IVH)	5 (5.10%)	3 (3.06%)	0.721
Late onset sepsis (LOS)	47 (47.96%)	39 (39.80%)	0.314
Days of hospital stay	62.00 (52.00 ~ 77.00)	60.00 (44.75 ~ 73.25)	0.163

Note: The continuous data were presented as median (25%~75% percentile) and analyzed using Mann-Whitney test. Frequencies for categorical variables reported as n (%) between the 2 groups were determined by Fisher’ s exact test

### Comparison of serum 25(OH)D levels in preterm infants between the two groups at 36 weeks’ PMA

The data regarding serum levels of 25(OH)D at baseline and at 36 weeks’ PMA in both two groups did not exhibit a normal distribution after performing Shapiro-Wilk test (all *P* < 0.001). The median 25(OH)D serum levels in the nesting + vitamin D and the nesting groups at baseline were 13.70 ng/mL (IQR: 8.60 ~ 19.13 ng/mL) and 14.55 ng/mL (IQR: 8.80 ~ 19.30 ng/mL), respectively, showing no significant difference (*P* = 0.735, Mann-Whitney test). However, at 36 weeks’ PMA, higher median serum level of 25(OH)D was found in the nesting + vitamin D [38.40 ng/mL (IQR: 17.20 ~ 70.88) ng/mL] as compared to the nesting group [15.95 ng/mL (IQR: 10.80 ~ 24.30) ng/mL] using Mann-Whitney test (*P* < 0.001, Table 3). Furthermore, the proportion of infants with VDD [25(OH)D serum level < 20 ng/mL] were also comparable at baseline between the 2 groups (*P* = 0.588, Fisher’ s exact test). However, after intervention, the proportion of infants with VDD was significantly lower in the nesting + vitamin D group than in the nesting group [34.69% vs. 71.43%; relative risk (RR): 0.486; 95% CI: 0.360 ~ 0.655; *P* < 0.001, Fisher’ s exact test].

**Table 3:** Comparison of serum 25(OH)D levels and anthropometric parameters in preterm infants between the two groups at baseline and at 36 weeks’ postmenstrual age (PMA)

	Nesting (n = 98)	Nesting + vitamin D (n = 98)	*P*
Serum 25(OH)D (ng/mL)			
At baseline	13.70 (8.60 ~ 19.13)	14.55 (8.80 ~ 19.30)	0.735
At 36 weeks’ PMA	15.95 (10.80 ~ 24.30)	38.40 (17.20 ~ 70.88)	< 0.001
Head circumference (mm)			
At baseline	279.0 (269.5 ~ 285.3)	276.0 (263.8 ~ 286.3)	0.319
At 36 weeks’ PMA	314.0 (311.8 ~ 317.0)	320.0 (315.0 ~ 326.0)	< 0.001
Weight (g)			
At baseline	1387 (1250 ~ 1567)	1405 (1259 ~ 1588)	0.883
At 36 weeks’ PMA	2209 (2101 ~ 2315)	2373 (2215 ~ 2520)	< 0.001
Length (cm)			
At baseline	38.10 (36.23 ~ 38.90)	37.50 (36.00 ~ 38.85)	0.370
At 36 weeks’ PMA	43.90 (43.00 ~ 44.60)	44.40 (43.50 ~ 45.40)	< 0.001
Body mass index (BMI, kg/m2)			
At baseline	10.24 (9.23 ~ 10.92)	10.16 (9.35 ~ 10.81)	0.815
At 36 weeks’ PMA	11.49 (10.88 ~ 12.12)	12.04 (11.33 ~ 12.78)	< 0.001

Note: The data were presented as median (25%~75% percentile) and analyzed using Mann-Whitney test

### Comparison of physical growth in preterm infants between the two groups at 36 weeks’ PMA

The data regarding parameters of physical growth at baseline and at 36 weeks’ PMA in both two groups did not exhibit a normal distribution after performing Shapiro-Wilk test, including head circumference, length, weight and BMI (all *P* < 0.001). No significance in these parameters, including length, weight, BMI and head circumference was found at baseline between the nesting group and the nesting + vitamin D group (all *P* > 0.05). However, these anthropometric parameters were significantly improved in the nesting + vitamin D group after intervention as compared to the nesting group (Table 3). In detail, the infants in the nesting + vitamin D group showed increased median head circumference [320.0 mm (IQR: 315.0 ~ 326.0 mm) vs. 314.0 mm (IQR: 311.8 ~ 317.0 mm)], weight [2373 g (IQR: 2215 ~ 2520 g) vs. 2209 g (IQR: 2101 ~ 2315 g)], length [44.4 cm (IQR: 43.5 ~ 45.4 cm) vs. 43.9 cm (IQR: 43.0 ~ 44.6 cm)], and BMI [12.04 kg/m^2^ (IQR: 11.33 ~ 12.78 kg/m^2^) vs. 11.49 kg/m^2^ (IQR: 10.88 ~ 12.12 kg/m^2^)] than the nesting group (Mann-Whitney test, all *P* < 0.001).

### Comparison of neurologic development in preterm infants between the two groups at 36 weeks’ PMA

As for the results of the neurodevelopment assessment (Table 4), the comparison of 3 categories (neurological, movement and responsiveness) in infants between the nesting group and nesting + vitamin D group showed statistically significant difference (Mann-Whitney test). The median values of neurological score [32.0 (IQR: 28.0 ~ 34.0) vs. 30.0 (IQR: 26.0 ~ 32.5), *P* = 0.033], movement score [34.0 (IQR: 32.0 ~ 36.0) vs. 32.0 (IQR: 30.0 ~ 34.0), *P* = 0.007], and responsiveness score [32.0 (IQR: 30.0 ~ 34.0) vs. 30.0 (IQR: 28.0 ~ 32.0), *P* = 0.024] were higher in infants from the nesting + vitamin D group than those in the nesting group. Moreover, the total score showed a significantly lower in the nesting group compared to the nesting + vitamin D group [94.0 (IQR: 87.5 ~ 98.0) vs. 96.0 (IQR: 93.5 ~ 100.5), *P* < 0.001].

**Table 4:** Comparison of neurodevelopment in preterm infants between the two groups at 36 weeks’ postmenstrual age (PMA)

Premie-Neuro (PN)	Nesting (n = 98)	Nesting + vitamin D (n = 98)	*P*
Neurological	30.0 (26.0 ~ 32.5)	32.0 (28.0 ~ 34.0)	0.033
Movement	32.0 (30.0 ~ 34.0)	34.0 (32.0 ~ 36.0)	0.007
Responsiveness	30.0 (28.0 ~ 32.0)	32.0 (30.0 ~ 34.0)	0.024
Total	94.0 (87.5 ~ 98.0)	96.0 (93.5 ~ 100.5)	< 0.001

Note: The data were presented as median (25%~75% percentile) and analyzed using Mann-Whitney test

## Discussion

Currently, there was no consensus recommendation for the dose of vitamin D in preterm infants, but some previous researchers have showed the advantage of daily 400 IU vitamin D_3_ supplementation as adequate for bone health in preterm and full-term infants [26]. For example, a systematic review and meta-analysis of clinical intervention trials by Zittermann A et al. revealed that 400 IU/day Vitamin D supplementation was sufficient for achieving 25(OH)D concentrations in infants, thus being able to prevent nutritional rickets [27]. Moreover, preterm infants receive 400, 800 or 1000 IU/day of vitamin D_3_ did not differ in anthropometric measurements and mortality according to the result of a randomized controlled trial [28] and an updated meta-analysis [29]. We firstly showed that a smaller percentage of VDD at 36 weeks’ PMA in nesting + vitamin D group in infants daily allocated to 400 IU vitamin D according to our policy. Besides, as compared to the nesting group, the proportion of infants with VDD was significantly lower in the nesting + vitamin D group (RR = 0.486; 95%CI: 0.360 ~ 0.655) at 36 weeks’ PMA with increased serum 25(OH)D concentration, suggesting that implementing vitamin D supplementation did significantly decrease the incidence of VDD in a population of preterm infants. As compared to the data at baseline in the nesting group, patients with VDD decreased at 36 weeks’ PMA without significance, which may be affected by small simple size.

The importance of nesting position with the flexion posture for the well‑being of the infant has been documented, which is an intrauterine position to support infants in mid line, thus improving sleep and development of sensory systems with alleviating pain during painful procedure [30]. To stabilize body posture, supine nesting position has been adopted by most NICUs in worldwide, and that was the reason for this position in the present study [31, 32]. As the gold standard for promoting comfort of hospitalized preterm infants, nesting is a favoring factor for the baby’s sleep when compared to its non-use by a previous study [33], which could conserve energy and minimize weight loss [34]. In our study, the infants showed increased physical growth, including length, weight, BMI and head circumference after the nesting intervention, which was further increased in infants after the combination of nesting intervention and vitamin D supplementation. Several studies have been reported that vitamin D supplementation during pregnancy improves infant birth weight with increased infant length [35–37]. All mentioned above indicated vitamin D supplementation in NICU could obviously improve the physical growth in preterm infants who received nesting intervention.

Preterm infants, particularly those at young PMAs, are high risk for delayed neurodevelopment [38], emphasizing the importance of clinical neurologic examination for the evaluating the neonate’s progress [39]. Premie-Neuro (PN), a standardized neurologic assessment tool with good construct validity, has been widely used for preterm infants [24]. A previous study demonstrated that high-risk infants with a discharge Neurobehavioral Rating Scale of less than 5 had lower PN scores than low-risk infants, being consistent with research findings using other assessment tools, such as the Test of Infant Motor Performance [25]. Vitamin D is essential for several physiological functions and biological processes, and emerging evidences suggested newborn vitamin D levels was involved in brain development and was closely correlated with intellectual disability [40, 41]. In our study, the categories of PN, including neurological, movement and responsiveness scores, were higher in infants from the nesting + vitamin D group than those in the nesting group with higher total score, suggesting the beneficial effect of vitamin D supplementation in neurologic development of preterm infants receiving nesting care in the NICU.

One of the limitations of the study is that the duration of vitamin D supplementation as a potential factor causing result and conclusion bias should be considered in the future because the enrolled newborns between 28 ~ 32 weeks of GA and the assessment of physical growth and neurologic development at 36 weeks’ PMA. Moreover, as the retrospective nature of this study, the reasons for the infants not receiving additional vitamin D supplement in the nesting group was not analyzed. Therefore, this was possible confounding variable in the study. In addition, complete description of cohort more data should be further analyzed because some of them might have the influence on the observed outcomes.

In conclusion, vitamin D supplementation (400IU) effectively decreased the prevalence of VDD and led to higher concentrations of 25(OH)D at 36 weeks PMA in preterm-born newborns receiving nesting in the NICU, accompanying by the improved physical growth (weight, length, BMI and head circumference) and neurologic development (PN score). To the best of our knowledge, we were the first to report a novel approach using vitamin D supplementation in preterm infants who were given nesting care in the NICU. As both groups received nesting intervention, this was one more study that supported the necessity of vitamin D supplementation to improve physical growth and neurologic development of preterm infants.

## Data Availability

The datasets used and/or analysed during the current study are available from the corresponding author on reasonable request.
